# Self-cleaning semiconductor heterojunction substrate: ultrasensitive detection and photocatalytic degradation of organic pollutants for environmental remediation

**DOI:** 10.1038/s41378-020-00222-1

**Published:** 2020-12-28

**Authors:** Mingyue Hu, Yingnan Quan, Shuo Yang, Rui Su, Huilian Liu, Ming Gao, Lei Chen, Jinghai Yang

**Affiliations:** 1 Key Laboratory of Functional Materials Physics and Chemistry of the Ministry of Education, Jilin Normal University, 130103 Changchun, People’s Republic of China; 2 National Demonstration Centre for Experimental Physics Education, Jilin Normal University, 136000 Siping, People’s Republic of China; 3 Key Laboratory of Preparation and Application of Environmental Friendly Materials, Jilin Normal University, Ministry of Education, 130103 Changchun, People’s Republic of China; 4 College of Science, Changchun University, 130022 Changchun, People’s Republic of China; 5 Changchun Institute of Optics, Fine Mechanics and Physics, Chinese Academy of Sciences, 130103 Changchun, People’s Republic of China

**Keywords:** Applied optics, Nanoscience and technology, Nanoscale materials

## Abstract

Emerging technologies in the field of environmental remediation are becoming increasingly significant owing to the increasing demand for eliminating significant amounts of pollution in water, soil, and air. We designed and synthesized MoS_2_/Fe_2_O_3_ heterojunction nanocomposites (NCs) as multifunctional materials that are easily separated and reused. The trace detection performance of the prepared sample was examined using bisphenol A (BPA) as the probe molecule, with limits of detection as low as 10^−9^ M; this detection limit is the lowest among all reported semiconductor substrates. BPA was subjected to rapid photocatalytic degradation by MoS_2_/Fe_2_O_3_ NCs under ultraviolet irradiation. The highly recyclable MoS_2_/Fe_2_O_3_ NCs exhibited photo-Fenton catalytic activity for BPA and good detection ability when reused as a surface-enhanced Raman scattering (SERS) substrate after catalysis. The SERS and photocatalysis mechanisms were proposed while considering the effects of the Z-scheme charge-transfer paths, three-dimensional flower-like structures, and dipole–dipole coupling. Moreover, the prepared MoS_2_/Fe_2_O_3_ NCs were successfully applied in the detection of BPA in real lake water and milk samples. Herein, we present insights into the development of MoS_2_/Fe_2_O_3_ materials, which can be used as multifunctional materials in chemical sensors and in photocatalytic wastewater treatments for the removal of recalcitrant organic pollutants.

## Nanomaterials: environmental remediation

Photocatalytic MoS_2_/Fe_2_O_3_ heterojunction nanocomposites are able to remove organic pollutants from wastewater under ultraviolet light. Material systems are needed that can remove organic pollutants from the environment, many of which are driven by photocatalysis. As well as the challenge of efficiently removing contaminants from the environment, the photocatalytic material must also be recovered afterwards. Here, a team led by Jinghai Yang from Jilin Normal University report a MoS_2_/Fe_2_O_3_ nanocomposite that photocatalytically degrades bisphenol A under ultraviolet irradiation. Their semiconducting composite can also function as an effective SERS catalysis for bisphenol A. Compared to the component materials – MoS_2_ and Fe_2_O_3_ – the rate of degradation and sensitivity of the nanocomposite were both enhanced, and could be recovered from a solution via the use of a magnetic field.

## Introduction

Serious environmental pollution and accelerated global warming are attributed to the rapid consumption of fossil fuels, the increasing population, and the rapid development of the economy. Thus, the development of innovative and renewable environmental remediation materials is becoming increasingly important^1–6^. Since mechanically exfoliated graphene was discovered, the development of two-dimensional (2D) materials consisting of atomically thin crystal layers bound by van der Waals forces has accelerated owing to the potential applications of these materials in optoelectronics, catalysis, new technologies, and electricity^7–9^. 2D-MoS_2_ nanosheets are excellent layered materials, having unique layered structures and large surface areas. It is important to investigate methods for improving the chemical properties of MoS_2_, which may affect its application in electronic devices, catalysis, and molecular sensing^10^. A popular method for improving the properties of MoS_2_ is the decoration of MoS_2_ with noble metal nanoparticles. For instance, a MoS_2_/noble metal nanoparticle composite can induce local surface plasmon resonance (LSPR) for activating the photoelectrocatalysis of H_2_ and enhancing the light absorption or emission of MoS_2_. Moreover, the LSPR can generate surface-enhanced Raman scattering (SERS), which can be used in biological and chemical sensing applications^11–13^. Among various traditional noble metal materials, Au nanomaterials are the most widely used SERS substrate material^14–16^. However, the high cost and specialized instruments required for Au substrates hinder their practical application. Due to its very high SERS activity, Ag is another widely studied substrate material^17^. Although the price of Ag is much lower than the price of Au, the main defect of Ag is its poor stability, which easily oxidizes in air. To address these problems, it is essential to exploit synergistic effects by incorporating inexpensive and stable semiconductors.

Thus far, a few MoS_2_-based heterostructures, such as CdS/MoS_2_, TiO_2_/MoS_2_, and MoO_3_/MoS_2_, have exhibited higher photocatalytic efficiencies than pristine MoS_2_^18–20^. Investigations have been continuously conducted on the efficient separation of a nanocomposite (NC) from a treated effluent, along with the subsequent reusability of the NC. Several research groups have begun to focus on magnetically separable photocatalysts for wastewater treatment, demonstrating the value of the special properties of magnetic materials. Among these magnetic materials, Fe_2_O_3_ has a narrow bandgap, high chemical resistance, and high resistance to corrosion. Therefore, rationally designed MoS_2_/Fe_2_O_3_ NCs can serve as a reusable SERS substrate for detection and easily reclaimed photocatalyst. The recovery and economical reuse of MoS_2_/Fe_2_O_3_ NCs photocatalysts is easily achieved by adding an external magnetic field.

Bisphenol A (BPA) is believed to be an endocrine disruptor and widely exists in food containers and the environment. Even low levels of BPA entering the body can disrupt the endocrine system by binding to estrogen receptors, which may lead to cardiovascular diseases, immune function deficiencies, impaired reproductive capacity, and other diseases^21–23^. Thus, it is imperative to develop a facile, rapid, and inexpensive method for BPA detection and degradation.

In this study, MoS_2_/Fe_2_O_3_ NCs were prepared via a simple low-temperature hydrothermal method, and the advantages of the two materials were combined. For example, after 50 min of ultraviolet (UV) irradiation, the substrate completely degraded BPA, and upon recovery, demonstrated its detection capability. Compared with MoS_2_ NFs and Fe_2_O_3_ NPs, the rate of degradation of BPA and the SERS activity of MoS_2_/Fe_2_O_3_ NCs were significantly better. This new, easily recoverable SERS sensor with a high sensitivity will facilitate sensing harmful molecules. To the best of our knowledge, no MoS_2_/Fe_2_O_3_ composites that exhibit BPA detection and photocatalysis multifunctionality have been reported thus far. Photocatalytic and SERS mechanisms were also proposed.

## Results and discussion

### Characterization analysis of MoS_2_/Fe_2_O_3_ NCs

**Fig. 1:**
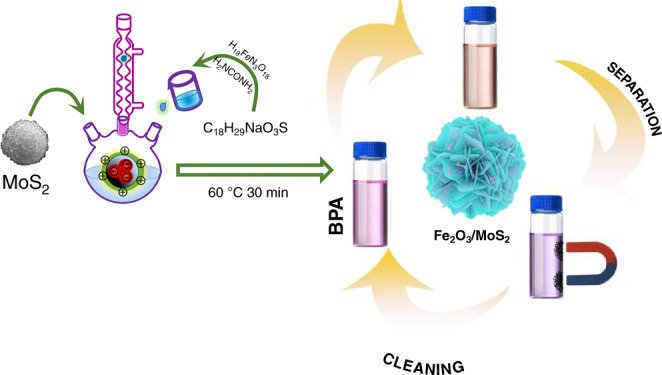
Preparation process and application of MoS_2_/Fe_2_O_3_Cs. Schematic showing the preparation process and application of MoS_2_/Fe_2_O_3_ NCs.

**Fig. 2:**
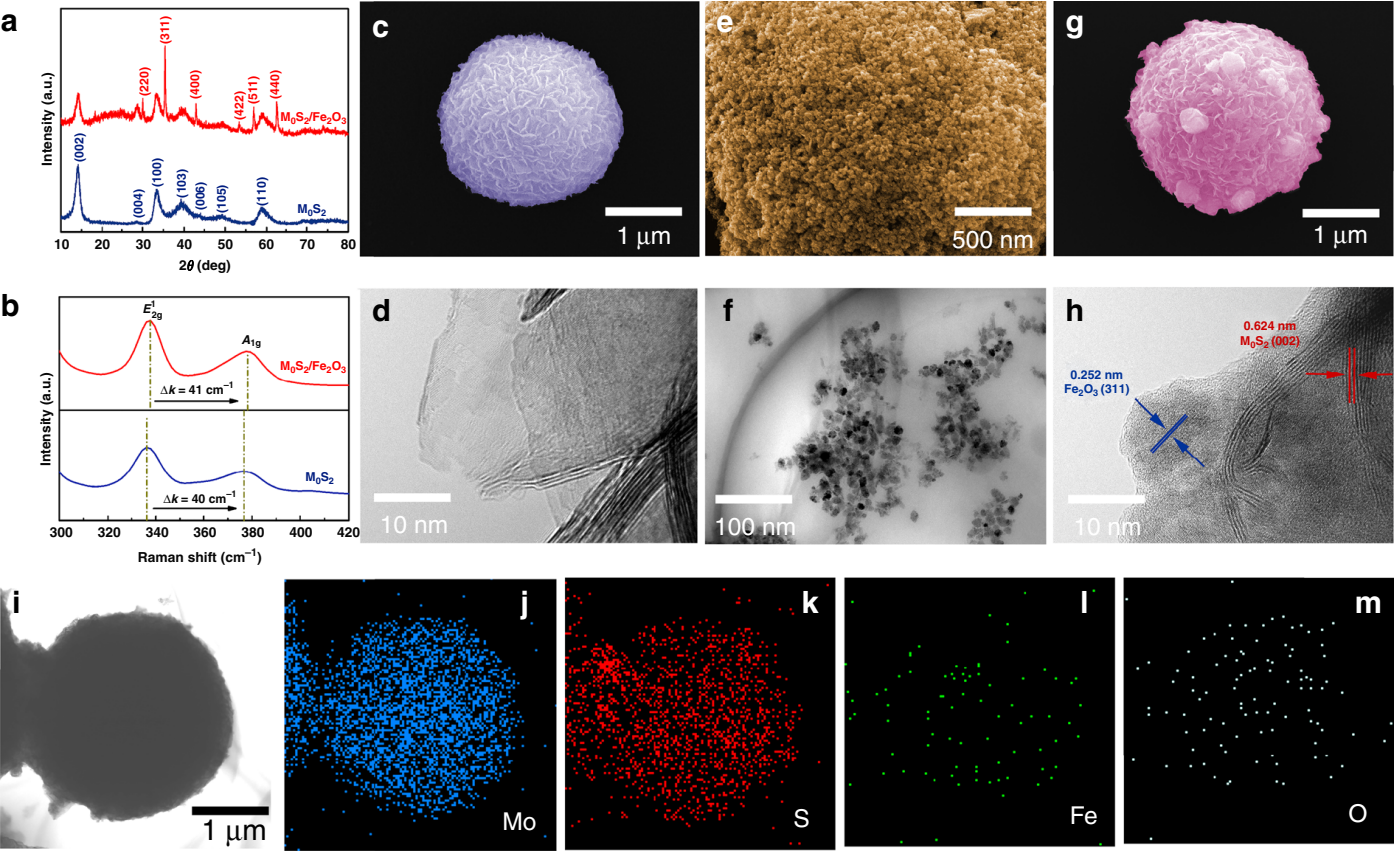
Structural quality and morphology of MoS_2_/Fe_2_O_3_ NCs. **a** XRD patterns of MoS_2_ and MoS_2_/Fe_2_O_3_. **b** Raman spectra of MoS_2_ and MoS_2_/Fe_2_O_3_. **c** SEM images of MoS_2_ NFs. **d** TEM image of typical MoS_2_ NFs. **e** SEM image of Fe_2_O_3_ NPs. **f** TEM image of the Fe_2_O_3_ NPs. **g** SEM image of MoS_2_/Fe_2_O_3_ NCs. **h** HRTEM image of MoS_2_/Fe_2_O_3_ NCs. **i**–**m** Typical STEM image of MoS_2_/Fe_2_O_3_ and the corresponding elemental mapping images of Mo, S, Fe, and O.

A growth flow diagram of the MoS_2_/Fe_2_O_3_ NCs is shown in Fig. 1. Figure 2a confirms that the MoS_2_ sample was pure hexagonal 2H-MoS_2_ (JCPDS card no. 37-1492). The peak with the highest intensity (at 2*θ* = 14.09°) indicated that MoS_2_ had excellent lamellar growth in the *c*-axis direction. In regard to MoS_2_/Fe_2_O_3_, some of the peaks corresponded to 2H-MoS_2_, while others corresponded to tetragonal γ-Fe_2_O_3_ (JCPDS card no. 39-1346) phase, indicating that the native structure of each constituent was well preserved during the reaction. The intensities of the MoS_2_ peaks for the MoS_2_/Fe_2_O_3_ NCs were lower than those for pure MoS_2_ because the Fe_2_O_3_ NPs attached to the MoS_2_ nanoflowers (NFs). Raman spectra confirmed the chemical composition of the MoS_2_/Fe_2_O_3_ NCs and MoS_2_. Two characteristic Raman peaks of MoS_2_ were observed at 337 and 377 cm^−1^, corresponding to the *A*_1g_ and ^1^*E*_2g_ vibration modes, respectively; additionally, their peak frequency difference was Δ*k* = 40 cm^−1^ (Fig. 2b)^24–26^. However, after the incorporation of Fe_2_O_3_, the characteristic Raman peaks of MoS_2_ shifted to 338 and 379 cm^−1^, and the peak frequency difference was Δ*k* = 41 cm^−1^. Δ*k* represents the number of MoS_2_ layers^27^. As shown in Fig. 2c, the pristine MoS_2_ samples were flower-like nanospheres with diameters of ~1–2 μm. Wrinkles and scrolling were observed in the transmission electron microscopy (TEM) images (Fig. 2d), indicating the extremely small thickness of the 2D structure. Scanning electron microscope (SEM) (Fig. 2e) revealed the presence of Fe_2_O_3_ nanoparticles (well below 20 nm in size according to TEM, Fig. 2f). As shown in Fig. 2g, Fe_2_O_3_ nanoparticles were dispersed, and a few Fe_2_O_3_ nanoparticle aggregations were present on the MoS_2_ NF. The high-resolution TEM image in Fig. 2h provided further insight regarding the morphology and microstructure of MoS_2_/Fe_2_O_3_. The *d*-spacing of the lattice stripes of Fe_2_O_3_ was 0.252 nm, which corresponded to the (0 0 1) lattice plane of hexagonal Fe_2_O_3_^28^. In regard to the MoS_2_/Fe_2_O_3_ NCs, the lattice spacing was 0.624 nm, corresponding to the hexagonal MoS_2_ (0 0 2) plane. In addition, the boundary between Fe_2_O_3_ and MoS_2_ was clearly observed, indicating that a heterojunction was formed between these two components. The regions with different colors in Fig. 2j–m correspond to S, Mo, Fe, and O, and the elemental distribution in MoS_2_/Fe_2_O_3_ was uniform.

**Fig. 3:**
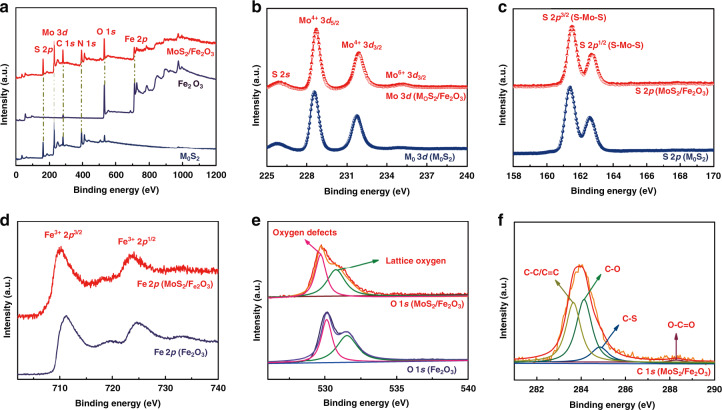
The electronic states and chemical composition of the MoS_2_/Fe_2_O_3_ NCs. **a** XPS survey spectra for MoS_2_/Fe_2_O_3_ NCs, MoS_2_, and Fe_2_O_3_. XPS spectra of MoS_2_/Fe_2_O_3_ and MoS_2_: **b** Mo 3*d*, **c** S 2*p*, **d** Fe 2*p*, **e** O 1*s*, and **f** C 1s of the MoS_2_/Fe_2_O_3_ NCs.

X-ray photoelectron spectroscopy (XPS) was performed to analyze the electronic states and chemical composition of the MoS_2_/Fe_2_O_3_ NCs (Fig. 3). The survey scan spectra of pristine MoS_2_, Fe_2_O_3_, and MoS_2_/Fe_2_O_3_ NCs are presented in Fig. 3a, which confirmed the coexistence of Fe 2*p*, O 1*s*, Mo 3*p*, and S 2*p* in the hybrid. The Mo 3*d* spectra exhibited three peaks for pristine MoS_2_, but after forming the MoS_2_/Fe_2_O_3_ NCs, four peaks appeared in Fig. 3b. The peaks at 235.8, 232.6, 229.4, and 226.5 eV corresponded to Mo^6+^ 3*d*_3/2_, Mo^4+^ 3*d*_3/2_, Mo^4+^ 3*d*_5/2_, and S 2*s*, respectively. A small portion of Mo^4+^ was oxidized into Mo^6+^ during the reaction, confirming that Fe_2_O_3_ was successfully recombined with MoS_2_. In Fig. 3b, the two peaks at 163.3 and 162.2 eV could be assigned to the doublet S 2*p*_1/2_ and S 2*p*_3/2_ orbitals of divalent sulfide ions (S^2^^−^), respectively, in agreement with the formation of the MoS_2_ nanostructure^29^. The Fe 2*p* spectrum exhibited two peaks at 710.4 and 723.7 eV (Fig. 3c), corresponding to the Fe 2*p*_3/2_ and Fe 2*p*_1/2_ components of γ-Fe_2_O_3_^30^. XPS peak shifts were also observed in the MoS_2_/Fe_2_O_3_ composites compared with pristine Fe_2_O_3_, confirming the successful formation of an electronically coupled interface between MoS_2_ and Fe_2_O_3_^31,32^. The high-resolution O 1*s* spectrum of the MoS_2_/Fe_2_O_3_ NCs is shown in Fig. 3d. The spectrum could be deconvoluted into two components: the peak at 529.3 eV was related to the binding energy of Fe-O in Fe_2_O_3_ caused by lattice O, and the peak at 530.7 eV was related to the O^2−^ ions in the anoxic region^33^. Specifically, the C 1*s* spectrum (Fig. 3f) was deconvoluted into four peaks at 283.7, 284.1, 284.9, and 288.3 eV corresponding to the C-C/C = C, C-O, C-S, and O-C = O bonds, respectively^34^.

### SERS enhancement and reusability of MoS_2_/Fe_2_O_3_ NCs for BPA detection

**Fig. 4:**
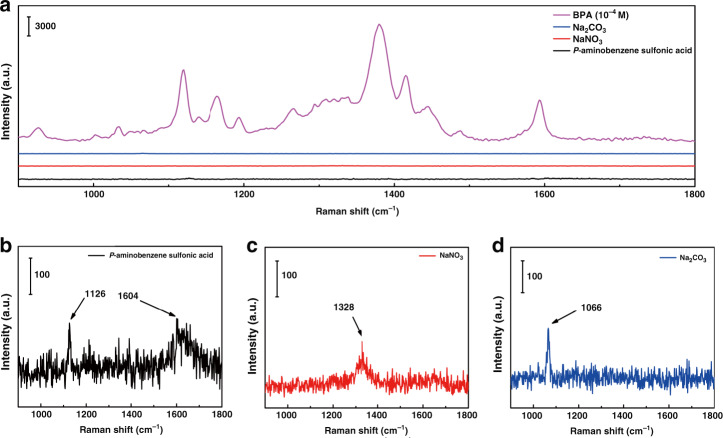
Detection of Pauly’s reagent. **a** SERS spectra of BPA, *p*-aminobenzene sulfonic acid, NaNO_3_, and Na_2_CO_3_ adsorbed on the MoS_2_/Fe_2_O_3_ NCs. **b** SERS spectrum of *p*-aminobenzene sulfonic acid adsorbed on the MoS_2_/Fe_2_O_3_ NCs. **c** SERS spectra of NaNO_3_ adsorbed on the MoS_2_/Fe_2_O_3_ NCs. **d** SERS spectrum of Na_2_CO_3_ adsorbed on the MoS_2_/Fe_2_O_3_ NCs.

**Fig. 5:**
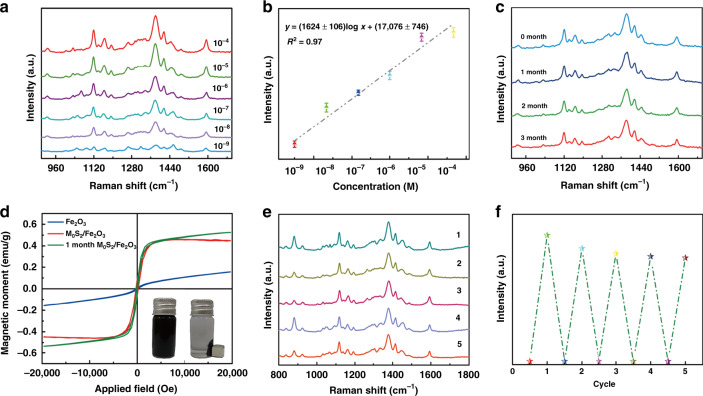
Sensitivity, stability, and reproducibility of MoS_2_/Fe_2_O_3_ NCs. **a** SERS spectra of MoS_2_/Fe_2_O_3_ NCs incubated with a BPA aqueous solution at various concentrations. **b** Calibration curve for BPA at 1124 cm^−1^. **c** SERS spectra of the 10^−4^ M BPA aqueous solution based on the MoS_2_/Fe_2_O_3_ substrate collected at different shelf times. **d** Room temperature magnetic hysteresis curves of the MoS_2_/Fe_2_O_3_ NCs and MoS_2_ NFs. The inset shows the water dispersibility and magnetic separability of the MoS_2_/Fe_2_O_3_ NCs. **e** SERS spectra of BPA after the self-cleaning test. **f** Corresponding normalized Raman intensities of 1385 cm^−1^ when the SERS substrate was recycled five times for the detection of 10^−4^ M BPA.

Herein, *p*-aminobenzene sulfonic acid, NaNO_3_, and Na_2_CO_3_ as Pauly’s reagent were added in the BPA detection test to enhance the adhesion of BPA on the surface of the MoS_2_/Fe_2_O_3_ NCs. As shown in Fig. 4, *p*-aminobenzene sulfonic acid, NaNO_3_ and Na_2_CO_3_, were all low Raman scattering active molecules; therefore, their addition had almost no effect on BPA detection. To confirm that MoS_2_/Fe_2_O_3_ had excellent SERS properties, SERS spectra of BPA absorbed on MoS_2_/Fe_2_O_3_ at various concentrations ranging from 10^−4^ to 10^−9^ M were obtained, as shown in Fig. 5a. These results indicated that as the concentration of BPA decreased, the intensity of the Raman peaks decreased. The characteristic peak of BPA at 1124 cm^−1^ was observed at concentrations as low as 10^−9^ M, indicating that the MoS_2_/Fe_2_O_3_ NCs had a high sensitivity. The intensity of the peak at 1124 cm^−1^ was correlated with the BPA concentration; thus, we used it for further quantitative analysis. Figure 5b shows the direct proportionality between the BPA concentration, in the range of 10^−4^–10^−9^ M, and the normalized Raman signal intensity. The linear equation is as follows:

1 $${\mathrm{log}}(I_{1124}) = (1624 \pm 106){\mathrm{log}}\,C_{\mathrm{BPA}} + (17,076 \pm 746)$$

with a squared correlation coefficient of *R*^2^ = 0.97. The stability of the substrate is an important factor that must be considered. As shown in Fig. 5c, the SERS spectrum of the MoS_2_/Fe_2_O_3_ NCs substrate hardly changed over time; thus, the MoS_2_/Fe_2_O_3_ NCs could be stored for at least 3 months under ambient conditions.

As shown in Fig. 5d, the hysteresis loop of the MoS_2_/Fe_2_O_3_ NCs indicated excellent superparamagnetic behavior. The saturation magnetization (*M*_s_) value of the MoS_2_/Fe_2_O_3_ NCs (0.46 emu/g) was higher than that of Fe_2_O_3_ alone. This good magnetic property completely satisfied the requirements for magnetic separation. The uniformly dispersed MoS_2_/Fe_2_O_3_ NCs quickly separated from the solution and formed aggregates within 22 s when external magnets were used. Conversely, when the magnets were removed, the agglomerated MoS_2_/Fe_2_O_3_ quickly redistributed into the solution via slight shaking, as shown in the inset of Fig. 5d. In addition, we measured the magnetization of the MoS_2_/Fe_2_O_3_ NCs after 3 months and found that the *M*_s_ value hardly changed (from 0.46 to 0.52 emu/g). Hence, we concluded that MoS_2_/Fe_2_O_3_ NCs were highly stable at room temperature and atmospheric pressure. To test the reusability of the MoS_2_/Fe_2_O_3_ NCs, we repeated the SERS experiment five times with the same sample. After each experiment, the MoS_2_/Fe_2_O_3_ NCs were separated from the solution using a magnet. Figure 5e shows the SERS spectrum of a MoS_2_/Fe_2_O_3_ substrate that was reused after absorbing the same concentration of BPA; the results indicated that the substrate had good reproducibility. As shown in Fig. 5f, the MoS_2_/Fe_2_O_3_ NC substrate had excellent SERS activity even after five recycling runs. Although the average Raman intensity decreased slightly, it satisfied the qualitative testing requirements for BPA. These results indicate that the MoS_2_/Fe_2_O_3_ NCs were reproducible and show promise as reusable substrate materials.

### Detection in “real-world” samples

**Fig. 6:**
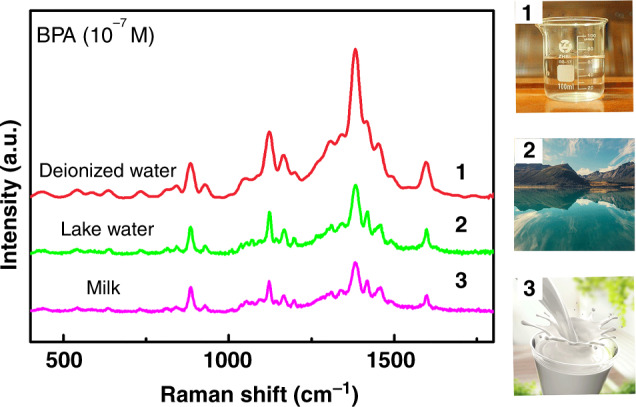
Detection of “real-world” samples. Detection of BPA using the MoS_2_/Fe_2_O_3_ NCs substrates in “real-world” samples.

To evaluate the application of the MoS_2_/Fe_2_O_3_ NCs, “real-world” samples (lake water and milk) were chosen for detection. As shown in Fig. 6, the characteristic CH wagging peak of BPA at 1124 cm^−1^ was observed at concentrations as low as 10^−7^ M for these samples, indicating that the MoS_2_/Fe_2_O_3_ NCs could be used for the practical and rapid detection of BPA.

### Photocatalytic activity of MoS_2_/Fe_2_O_3_ NCs

**Fig. 7:**
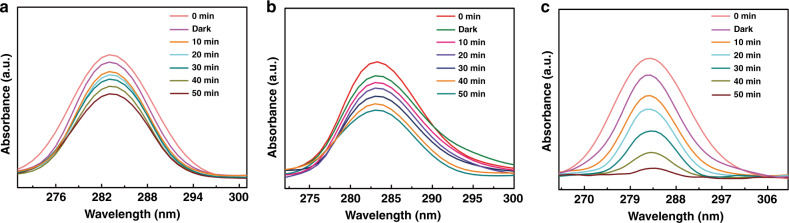
Photocatalytic activity of MoS_2_/Fe_2_O_3_ NCs. UV–vis absorption spectra of a BPA solution in the presence of **a** MoS_2_ NFs, **b** Fe_2_O_3_ NPs, and **c** MoS_2_/Fe_2_O_3_ NCs.

The catalytic properties of the pristine MoS_2_ NFs, Fe_2_O_3_ NPs, and MoS_2_/Fe_2_O_3_ NCs were evaluated by performing BPA degradation experiments under UV irradiation. The BPA degradation results for the MoS_2_ NFs, Fe_2_O_3_ NPs, and MoS_2_/Fe_2_O_3_ NCs samples under UV light are presented in Fig. 7a–c, respectively. For all catalysts, the intensity of the main absorption peak decreased with increasing irradiation time. After 50 min of UV irradiation, the degradation rates of the two pristine photocatalysts (MoS_2_ NFs and Fe_2_O_3_ NPs) were only ~40% and 48%, respectively. Surprisingly, the photocatalytic activity was significantly increased in the presence of the MoS_2_/Fe_2_O_3_ NCs catalyst; in this case, >92% of the present BPA was decomposed after 50 min of irradiation, as shown in Fig. 7c. This degradation rate is significantly higher than those observed with the MoS_2_ NFs and Fe_2_O_3_ NPs.

**Fig. 8:**
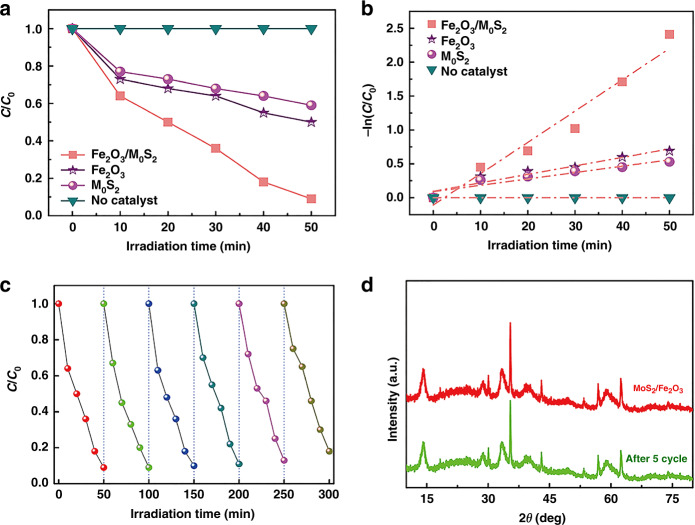
Photocatalytic activity and stable photoactivity of MoS_2_/Fe_2_O_3_ NCs. **a** Evolution of the relative concentrations (*C*/*C*_0_) of BPA with several photocatalysts as a function of irradiation time. *b* Corresponding kinetic plots of the photocatalytic degradation of BPA with the selected photocatalysts. **c** Reusability and stable activity of the MoS_2_/Fe_2_O_3_ NCs after being used for five cycles of the photocatalytic degradation of BPA. **d** XRD pattern of the MoS_2_/Fe_2_O_3_ NCs before and after the fifth cycle.

A related graph showing the dependence of the BPA degradation efficiencies of the MoS_2_/Fe_2_O_3_ NCs and other catalysts on the UV irradiation time is presented in Fig. 8a. We define the degradation efficiency as *C*/*C*_0_, where *C*_0_ represents the initial BPA concentration (mg/L) and *C* represents the BPA concentration after the reaction (mg/L). As shown in Fig. 8a, the MoS_2_/Fe_2_O_3_ NCs had better photocatalytic activity than the other catalysts. The photocatalytic efficiency of MoS_2_/Fe_2_O_3_ NCs was as high as 0.02, which was higher than that of pure MoS_2_ (0.01) and Fe_2_O_3_ (0.008). Thus, the MoS_2_/Fe_2_O_3_ NCs has great potential for use in wastewater treatments. Before light irradiation, the photocatalyst and BPA solution were stirred under dark conditions for 10 min to attain an adsorption equilibrium. During this period, the concentration of BPA decreased because of the adsorption of BPA molecules on the photocatalysts. We used the pseudo-first-order mode to investigate the reaction kinetics of BPA degradation. The simplified equation is:

2 $$- {\mathrm{ln}}(C/C_0) + kt$$

where *k* represents the apparent first-order reaction rate constant^35^. Figure 8b shows the relationship between −ln(*C*/*C*_0_) and the irradiation time for different photocatalysts. The curves could be fitted with a linear relationship, indicating that the degradation kinetics followed a typical first-order reaction. Using Eq. (1), we determined the apparent pseudo-first-order rate constants for the different photocatalysts. The *k* values of the pristine Fe_2_O_3_ NPs, MoS_2_ NFs, and MoS_2_/Fe_2_O_3_ NCs were calculated to be 0.69, 0.53, and 2.41, respectively. Stable photoactivity under UV light is critical for practical water treatment applications, particularly for composite materials that may lose their coating. We examined the loss of the BPA degradation activity of the MoS_2_/Fe_2_O_3_ NCs by utilizing it for five consecutive cycles under UV light irradiation. No loss of activity was observed (Fig. 8c). As shown in Fig. 8d, the structure of the catalyst was not significantly changed after five consecutive photocatalytic degradation cycles, also suggesting that the Fe_2_O_3_ nanoparticles could slow down the photocorrosion of MoS_2_, thereby efficiently protecting MoS_2_. Generally, MoS_2_ is prone to photocorrosion due to oxidation of surface sulfions to sulfurs by photoexcited holes. Therefore, the MoS_2_/Fe_2_O_3_ NCs exhibited high stability and excellent anti-photocorrosion properties, showing that this material has promise for use in environmental restoration applications.

### Mechanisms of SERS detection and photocatalysis

**Fig. 9:**
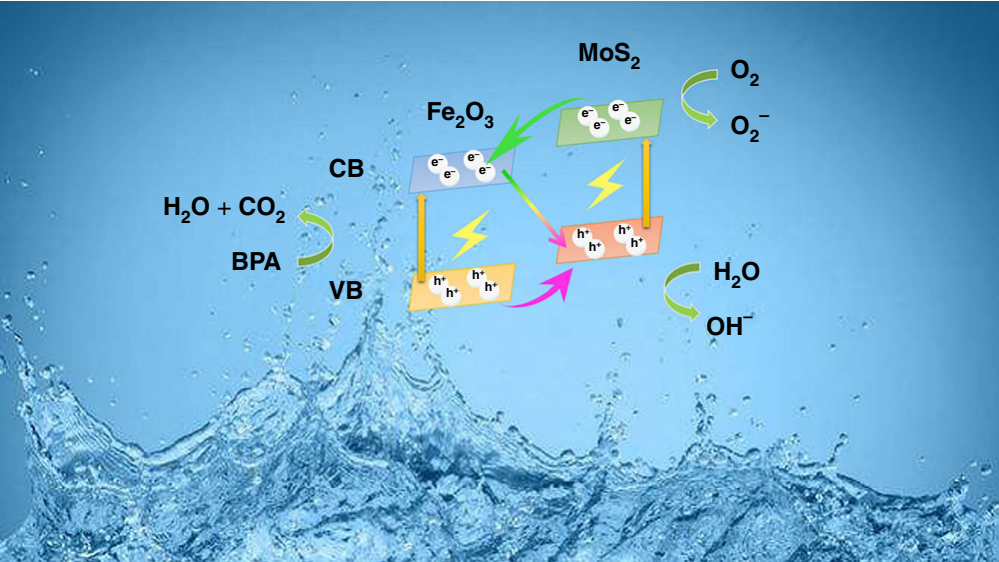
SERS and photocatalytic mechanisms of MoS_2_/Fe_2_O_3_NCs. Schematic illustration showing the proposed SERS and photocatalytic mechanisms of MoS_2_/Fe_2_O_3_ NCs.

When the MoS_2_/Fe_2_O_3_ heterojunction system was irradiated with UV light, MoS_2_ was excited, generating electron–hole pairs because of its narrow bandgap. The photoinduced electrons moved rapidly from the conduction band (CB) of MoS_2_ to that of Fe_2_O_3_, as shown in Fig. 9. In the MoS_2_/Fe_2_O_3_ NCs, the spatial separation of photoexcited holes and electrons extended the charge-carrier lifetime and hindered the recombination of electron–hole pairs, thereby enhancing the photocatalytic activity. Moreover, the selected transfer of holes from the valence band (VB) of MoS_2_ to Fe_2_O_3_ remarkably weakened the photocorrosion activity. After the carriers of MoS_2_ and Fe_2_O_3_ were generated, the free electrons accumulated in the CB of Fe_2_O_3_, while photoinduced holes were present in the VB of MoS_2_; thus, a high photocatalytic activity was obtained. Effective Z-type electron–hole pair separation and an effective transfer path were achieved, and a strong redox capacity of the photoexcited electron and holes was obtained in the CB and VB, respectively, significantly improving the photocatalytic and SERS activity of the MoS_2_/Fe_2_O_3_ NC heterojunction. Therefore, Fe_2_O_3_ not only acted as a protective shell for the MoS_2_ core by preventing the loss of sulfur but also constructed Z-type junctions that prolonged the lives of photogenerated electrons and holes, which would significantly enhance the photocatalytic activity and stability. Another reason for the SERS enhancement was the semiconducting nature of MoS_2_. Because its surface had S atoms and polar covalent bonds (Mo-S) perpendicular to the surface, this dipole–dipole coupling significantly increased the intensity of the Raman peaks^36^. In addition, because of the large surface-to-volume ratio, there was an abundance of active adsorption sites for gas molecules. The reactions involved in the photocatalytic process are summarized as follows:

3 $${\mathrm{Mo}}/{\mathrm{Fe}}_2{\mathrm{O}}_3 + h\nu \to {\mathrm{MoS}}_{\mathrm{2}}\left( {h^ + } \right) + {\mathrm{Fe}}_2{\mathrm{O}}_3\left( {{\mathrm{e}}^ - } \right)$$

4 $${\mathrm{O}}_{\mathrm{2}} + {\mathrm{e}}^ - \to {\dot{\mathrm O}}_2^ -$$

5 $${\dot{\mathrm O}}_2^ - + {\dot{\mathrm O}}_2^ - + 2{\mathrm{H}}^ + \to {\mathrm{H}}_2{\mathrm{O}}_2 + {\mathrm{O}}_2$$

6 $${\mathrm{H}}_{\mathrm{2}}{\mathrm{O}}_{\mathrm{2}} + {\mathrm{e}}^ - + h\nu \to {\dot{\mathrm{O}}{\mathrm {H}}} + {\mathrm{OH}}^ -$$

7 $${\mathrm{BPA}} + {\dot{\mathrm O}{\mathrm{H}}} \to {\mathrm{CO}}_2 + {\mathrm{H}}_2{\mathrm{O}} + {\mathrm{degraded}}\,{\mathrm{BPA}}$$

## Conclusion

In summary, a multifunctional material was fabricated by simply depositing Fe_2_O_3_ NPs onto MoS_2_ NFs, which significantly improved its photocatalytic properties and ability to be used as a SERS substrate. In addition, the MoS_2_/Fe_2_O_3_ NCs were successfully recycled. This study is the first to report MoS_2_/Fe_2_O_3_ NCs used as SERS substrates for BPA detection. The MoS_2_/Fe_2_O_3_ NCs had a detection limit of 1 × 10^−9^ M, along with exhibiting excellent stability. The prepared MoS_2_/Fe_2_O_3_ NCs had higher photocatalytic activity than the MoS_2_ NFs and Fe_2_O_3_ NPs alone. The enhanced photocatalytic activity and SERS activity were attributed to the efficient separation and transfer of electron–hole pairs by the Z-scheme heterojunction system. Therefore, as efficient multifunctional catalysts, MoS_2_/Fe_2_O_3_ NCs are expected to not only replace metal catalysts for removing organic matter from water and the environment but also pave the way for SERS applications, thereby introducing new methods for chemical and medical detection and for environmental monitoring.

## Materials and methods

### MoS2 NF preparation

First, 0.5 g H_4_MoNa_2_O_6_ and 0.7 g CH_4_N_2_S were mixed and stirred in 70 mL of ultrapure water. Then, 0.5 g C_6_H_8_O_7_·H_2_O was added until complete dissolution was achieved. The sample was transferred into an 80 mL Teflon-lined hydrothermal autoclave reactor and then placed in a drying box at 240 °C for 24 h. Next, the reaction products were centrifuged with alcohol and ultrapure water and dried at 70 °C.

### MoS_2_/Fe_2_O_3_ NC preparation

First, 0.2 g of MoS_2_ powder, 0.5 g of H_18_FeN_3_O_18_, and 0.7 g of H_2_NCONH_2_ were mixed in 70 mL of ultrapure water. Then, 0.02 g of C_18_H_29_NaO_3_S were well dispersed in the liquid mixture, stirred in a 60 °C water bath for 35 min, transferred to an 80 mL reactor, and finally placed in a drying box at 90 °C for 12 h. The MoS_2_/Fe_2_O_3_ NCs were washed with absolute ethanol and water to remove possible residuals. The solid powder solid was placed in a drying box and kept dry at 80 °C.

### Characterizations

XPS (ESCALAB250X, Thermo Scientific) and X-ray diffraction (XRD, D/Max 3C, Rigaku) were used to study the structural quality. TEM (JEM-2100HR, JEOL) and SEM (JSM-7800F, JEOL) were used to characterize the morphology of the samples. UV–visible absorption spectroscopy (UV-3600, Shimadzu Corporation) and a vibrating sample magnetometer (7407, Lake Shore) were used to characterize the optical and magnetic properties of the samples. Raman spectra were obtained with an Ar^+^-ion laser (inVia Raman, Renishaw).

### SERS experiments of BPA

We used the coupling reaction of BPA with Pauly’s reagents (*p*-aminobenzene sulfonic acid, HCl, NaNO_3_, and Na_2_CO_3_) to enhance the adhesion of BPA onto the surface of the SERS substrate materials. Please refer to our previous report for the detailed process^37^.

### SERS experiments of BPA in milk and lake water

Real milk contains fat, protein, vitamins, and other organic ingredients that can interfere with the detection of BPA. Therefore, it is necessary to pretreat the milk sample with BPA. The process is as follows. First, methanol (7 mL) and water (3 mL) were mixed and added to the milk sample (containing 10^−7^ M BPA, 4 mL), and then the mixture was sonicated and centrifuged at 10,000 r.p.m. for 3 min. The upper supernatant was extracted and then dried. This extract was collected in another centrifuge tube and mixed with methanol and water, with the above sonication and centrifugation process being repeated. Finally, the extract was filtered by membrane filters (0.45 and 0.22 µm) for the SERS test. The procedures for the detection and data analysis were the same as those for detecting BPA in water.

We collected lake water from a local source (South Lake in Changchun City). Lake water samples with BPA added were filtered by membrane filters (0.45 and 0.22 µm) before the detection test to avoid interference from other impurities. The test process was consistent with that described above.

### Photodegradation experiments

In the degradation process, circulating water was used to ensure that all the tests were performed at room temperature. The photodegradation of BPA under UV light was performed to assess the activity of photocatalysts. One hundred milliliters of an aqueous solution was prepared with 0.001 g of BPA and 0.05 g of Fe_2_O_3_/MoS_2_, MoS_2_, or Fe_2_O_3_ NCs. The test solution was stirred magnetically in a 100-mL beaker. The sample was kept in a dark room for 10 min for the adsorption of BPA molecules on the photocatalysts before being subjected to UV irradiation. During the experiment, the samples were taken at specified times. After each sampling, the catalyst was separated via centrifugation for testing.
